# Development and initial validation of a fertility experiences questionnaire

**DOI:** 10.1186/s12978-015-0054-3

**Published:** 2015-07-17

**Authors:** F. Scarlett Thomas, Joseph B. Stanford, Jessica N. Sanders, Shawn E. Gurtcheff, Mark Gibson, Christina A. Porucznik, Sara E. Simonsen

**Affiliations:** Office of Cooperative Reproductive Health, Division of Public Health, Department of Family and Preventive Medicine, University of Utah, 375 Chipeta Way Suite A, 84108 Salt Lake, UT USA; Utah Fertility Center, 1446 W. Pleasant Grove Blvd, 84062 Pleasant Grove, UT USA; Department of Obstetrics and Gynecology, University of Utah School of Medicine, Utah, USA

**Keywords:** Infertility, Questionnaires, Validation studies, Time to pregnancy

## Abstract

**Background:**

Many women throughout the world have history of subfertility (resolved or unresolved), but much remains unknown about services and treatments chosen.

**Methods:**

We developed a mixed-mode fertility experiences questionnaire (FEQ) in 2009 through literature review and iterative pilot work to optimize question format and mode of administration. The focus of the FEQ is to collect data retrospectively on time at risk for pregnancy, fertility treatments received and declined, pregnancy, time to pregnancy and pregnancy outcomes. We conducted a validation of key elements of the FEQ with comparison to medical records in 2009 and 2010. The validation sample was selected from women initially seen at a specialized fertility treatment center in Utah in 2004.

**Results:**

The FEQ was optimized with two components: 1) written (paper or web-based), self-administered, followed by 2) telephone- administered questions. In 63 patients analyzed, high levels of correlation were identified between patient self-report and medical records for the use of intrauterine insemination and assisted reproductive technology, pregnancy and live birth histories, time at risk for pregnancy and time to pregnancy. There was low correlation between medical records and self-report for the use of oral ovulation drugs and injectable ovulation drugs. Compared to the medical record, the FEQ was over 90 % sensitive for all elements, except injectable ovulation drugs (70 % sensitivity).

**Conclusions:**

The FEQ accurately captured elements of fertility treatment history at 5–6 years after the first visit to a specialty clinic.

**Electronic supplementary material:**

The online version of this article (doi:10.1186/s12978-015-0054-3) contains supplementary material, which is available to authorized users.

## Introduction

Subfertility (also called infertility) is a prevalent condition throughout the world [1, 2]. Based on the conventional definition of no pregnancy within a year of intercourse without contraception, the prevalence of subfertility is estimated at 15 % or more among couples currently trying to conceive in developed countries [2–4]. Many women or couples experiencing subfertility never seek services, and among those who do, the level of services received varies widely [5–7]. In addition to accessibility of medical services, demographic and social factors are strongly correlated with the types and volume of fertility treatments received [8, 9].

Questionnaires can provide valuable information on many aspects of subfertility, including its epidemiology, psychological aspects, and women’s and couple’s decisions about treatment. Such information is essential to inform efforts to improve medical treatment and to guide policy regarding the allocation of resources to diminish the societal and economic burden of subfertility [7, 10]. However, questionnaire research to date in the field has been largely ad hoc, and many questionnaires have not been validated.

Our goal was to generate a questionnaire instrument that can be used retrospectively to ascertain fertility treatments chosen by women, reasons for choosing or declining different treatments, factors that may have influenced choices of treatments, timing of treatments, and a detailed history of attempts to conceive (by which we mean time at risk for pregnancy, as defined further below). We conducted a validation comparing important components of the questionnaire with data from medical records in a clinical sample.

## Methods

### Design of the questionnaire

We initially conducted a literature review to identify questionnaires that possibly included domains of interest for our research [7, 11–20]. We also contacted authors to obtain copies of their instruments, where possible. We used items verbatim from some questionnaires [12, 13], and adapted items from others (as referenced in Table 1). Based on this review and consultation with experts in the field, we constructed a questionnaire with the domains of interest for our research, called here the fertility experiences questionnaire (*FEQ*).

**Table 1:** Domains, components, details and sources in the fertility experiences questionnaire

Domains^a^	Written component (paper or online)	Phone interview component	Details
General health [28]	X		Exercise, tobacco, caffeinated beverages, alcohol, past medical history, pap smear
Menstrual history	X		Age at menarche, frequency and intensity of menses (when not taking hormonal birth control or fertility treatment)
Sexual history	X		Number of lifetime sex partners, history of sexually transmitted infection
Pregnancies and attempts to conceive [23]	Definitions and list of attempts	Verification and detailed questions about attempts	Start month/year for “attempt,” how attempt started and ended, partner for attempt
Desire to conceive during each attempt [29]		X	Likert scale for desire for pregnancy and pereceived partner desire for pregnancy at beginning, middle and end of each attempt
Pregnancy outcomes [11]	Dates and types of outcomes	Verification and details	Live birth, miscarriage, ectopic, stillbirth, molar pregnancy, termination, currently pregnant, other, and date ended. For live birth: state where born, birth weight, sex, hospital stay of 7 days or more, breastfeeding.
Fertility-related medical evaluations^b^	X		Ultrasound of uterus/ovaries, follicular ultrasound, hysterosalpingogram, hysteroscopy, D&C, blood tests
Fertility-related surgeries^b^	X		C-section, cervical cryotherapy or LEEP, laparoscopy, laparotomy, surgical treatment of endometriosis, surgery on ovaries, tubes, or uterus, other abdominal or pelvic surgery, partner vasectomy reversal, partner other urologic surgery
Fertility-related diagnoses^b^	X		Unexplained infertility, endometriosis, PCOS, low progesterone or estrogen, not ovulating, abnormal ovulation, limited cervical mucus, pelvic adhesions, blocked fallopian tubes, uterine fibroids, uterine polyps, luteal phase defect, male factor, other
Fertility treatments recommended by physician or practitioner, and reasons for using or declining treatments	X	Details about treatments received, and linking timing to attempts to conceive, and whether linked to conception	Fertility-enhancing drugs, artificial insemination, in vitro fertilization with or without intracytoplasmic sperm injection, donor semen or donor eggs, acupuncture, fertility diets, herbal treatments
Self-help measures for trying to conceive (fertility awareness, diet, etc.)		Ascertained and linked to attempts to conceive, and whether linked to conception	Timed intercourse by counting days, basal body temperature, urine ovulation test kits, cervical mucus or fluid; took herbs, fertility vitamins, or supplements; lost weight; adhered to fertility diets; took a daily drug to enhance fertility; took a drug for ovulation; took hormones like progesterone
Adoption experiences	X		Ever applied for adoption, any adopted children
Stress and social situation [12]	X		Likert scale questions about impact of fertility problems and/or treatment on life, relationships with partner, family, friends; level of support from family, partner, friends; negative reactions from family, partner, friends.
Experience of past fertility treatment[12]	X		Likert scale questions about perceptions of past treatment: had enough time, shared decision making, feeling listened to, receiving explanations, addressing emotional issues
Demographic information	X		Marital status and date, education, race, ethnicity, country of birth, country of parents’ birth, languages spoken, religious preference, occupation, income, whether have written records of fertility experiences, best times to contact by phone
Friends and family with infertility	X		Number of friends or family diagnosed with infertility, friends or family members who have used any of the fertility measures listed previously above
Hypothetical interest in participating in studies of fertility treatment		X	Would she have been willing to participate in a study that would involve lifestyle advice, education about fertile days, herbs or acupuncture, medication, artificial insemination, or IVF.
Sources of information	X	X	Did the participant consult written records to complete the questionnaire?

^a^Citations indicate other studies from which sections of the questionnaire were taken or adapted

^b^Items in these sections of the questionnaire were adapted from questions used in research conducted by Mary Croughan, PhD, University of California, San Francisco

### Pregnancies and time at risk for pregnancy

We were particularly interested in assessing time at risk for pregnancy, pregnancies, and pregnancy outcomes retrospectively. The “pregnancies and attempts to conceive” component of the *FEQ* captures information about all time in a woman’s life when she was at risk of pregnancy, whether or not she was “trying” to conceive, and whether or not the “attempt” ended in pregnancy. Each time at risk for pregnancy is called an “attempt” to conceive in the *FEQ*. This written questionnaire contains a definition as well as an illustrative example for “attempts to conceive” to enhance respondents’ understanding of the concept of “attempts to conceive.” (See Additional file 1 for the full *FEQ*, which includes the exact definition and examples used in the online portion of the “Attempts to Conceive” section.) Several initial questions are asked in the written questionnaire, with a follow-up telephone interview for further verification and clarification. The goal is to capture as accurately as possible the time a woman was actually at risk of pregnancy. Unlike other time to pregnancy questions that ask a woman to give a general number of months it took her to become pregnant, the “attempts to conceive” section specifically excludes time that a woman was not at risk for pregnancy (for example, due to spousal separation, or desire not to have a baby in a certain month), even though she may have intended to achieve a pregnancy [21]. It also explicitly includes time that a woman was at risk of pregnancy without intending pregnancy [22]. Dates (month and year) are established for specific definitions of beginning an “attempt to conceive” and when the pregnancy occurred, similar to an approach previously used by European investigators in an interview questionnaire, but with some additional details [23]. For “attempts” that ended in pregnancy, there were additional questions about the outcome of the pregnancy.

### Other components of the *FEQ*

Other components of the *FEQ* addressed fertility evaluation and treatment, women’s general health, menstrual and sexual history, and psychological and social factors. All components of the *FEQ* are outlined in Table 1. The complete *FEQ* is in the Additional file 1.

### Pilot testing

We pilot tested the *FEQ* in four sequential phases: entirely by self-administration (written on paper, 10 women), entirely by face-to-face interview (5 women), entirely by telephone interview (3 women), and by mixed-mode of administration (written online, followed by interview, 30 women). All participants in the pilot testing were current patients of a specialty fertility clinic, the Utah Center for Reproductive Medicine (UCRM). The 18 women participating in the first three phases were a convenience sample from women being seen in the clinic during the time of the development work. The 30 women in the fourth phase were a random sample and were also included as the first group of women in the validation analysis, described in the next section below.

After each administration of the *FEQ*, a semi-structured debriefing interview was conducted (in person or by telephone) that explored which questions were perceived as difficult, confusing, or objectionable. Revisions of wording of some items and responses were undertaken after the first three phases. We also reviewed the time required to complete the *FEQ* and the consistency of responses in these phases. Oral interviews obtained information on time to conceive (as described above) that was more internally consistent than complete self-administration on paper. We also found that we had more complete and internally consistent information regarding pregnancy outcomes, fertility treatments, and self-help measures when these were assessed first in writing, with a follow-up telephone interview. Finally, we found that mixed-mode administration was more efficient than complete oral interviews and yielded internally consistent information on parts of the questionnaire that were not temporally tied to attempts to conceive.

### Validation

**Fig. 1:**
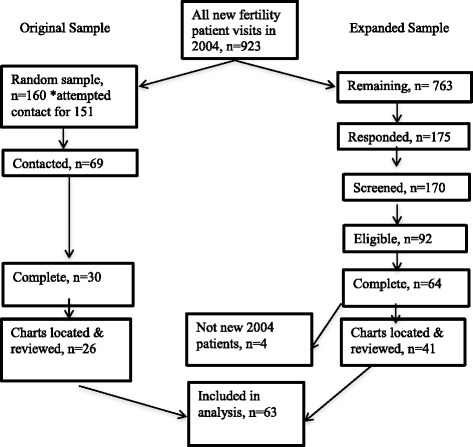
Recruitment and enrollment

This validation includes two groups of women. (See Fig. 1) The original group was selected via random sample of women over the age of 18 who had an initial consultation for subfertility generally, or in vitro fertilization (IVF) specifically at the UCRM in the year 2004. There were no other exclusion or inclusion criteria for this group. In 2009, we attempted to contact each woman by telephone three times at different times on a day, evening, and weekend to ask if she would be willing to receive information about the study. If she agreed, we obtained an e-mail address where we sent an e-mail describing this study. Included in the email was an attachment of a consent document as well as a link to the questionnaire and instructions to decline participation if she chose to do so. Women consented by clicking on the link to begin answering the questionnaire. Women who completed the online questionnaire were contacted by telephone for the follow-up interview at a time of their convenience to conduct the telephone component of the *FEQ*, the follow up interview typically occurred within 2 weeks of completion of the written survey. There was no compensation for these women.

A subsequent or expanded group was selected from the remainder of the UCRM patients who were also first seen for infertility or IVF in 2004; this group participated in a follow-on study that had additional eligibility criteria. These women all received a mailing in 2010 inviting them to contact the study investigators if they were interested in participation. Those who did so were screened for eligibility based on primary subfertility (defined by being at risk for pregnancy for 12 months with the same partner without pregnancy and never having a positive pregnancy test prior to being seen at the clinic) as well as living in Utah at the time of their appointment. These women received a $10 gift card for their participation.

For all participants, medical records were obtained from the UCRM for independent chart review to extract key variables for comparison. Review of medical records was done by two physicians at the UCRM (S.G., M.G.), and three medical students under their supervision. Records from any other clinics that patients may have visited in addition to UCRM were not available to us for analysis.

We used data from billing records to compare participants from both groups described above to all patients seen at the UCRM during 2004 (the source population, *n* = 923) on the characteristics of age, type of visit, and whether they had a subsequent visit for an early pregnancy ultrasound.

For the initial validation, we chose the following variables to compare between the medical chart review and the *FEQ*: use of oral ovulation enhancing drugs, use of injectable ovulation drugs, use of intrauterine insemination, use of in-vitro fertilization, time at risk for pregnancy, time to pregnancy, pregnancy, and live birth. These represent the outcomes of greatest interest for this questionnaire.

### Statistical analysis

We performed correlation analyses to determine the degree of agreement between the specified elements in the questionnaire and the patients’ medical records. For categorical variables (history of different types of treatment and pregnancy history), we calculated sensitivity and specificity, and used Cohen’s kappa statistic to rate interobserver agreement [24]. As a sensitivity analysis, we also repeated these analyses for the women who never conceived.

From the *FEQ* interview, we calculated time at risk for pregnancy starting at the woman’s report of the beginning of the first attempt to conceive. The end point for the time period was her first clinic visit to UCRM, which is also the time point that we extracted the time attempting to conceive that was recorded in the medical record. Between the start and end points, any time that was reported as not being at risk for pregnancy in the *FEQ* was subtracted from the interview-based time at risk for pregnancy, but not from the medical record-based time at risk for pregnancy. For women who had a pregnancy, we also calculated time to pregnancy. For this calculation, the same starting point was used from the *FEQ* interview, and the end point was the time of pregnancy, as reported by the woman. For the medical record, the time attempting to conceive that was recorded at the first visit was added to the subsequent time until the beginning of the first subsequent pregnancy identified in the medical record. To compare time at risk for pregnancy and time to pregnancy between the *FEQ* interview and the medical record, we used the Pearson’s linear correlation coefficient.

## Results

### Enrollment and sample characteristics

For the initial sample, we attempted to contact 160 women, randomly sampled from UCRM patients that were first seen in 2004 see (Fig. 1). Eighty-two (54 %) were not locatable. Of the 69 women (46 %) that we located, 61 (88 %) agreed to consider participating. Of these, 34 (56 %) completed the written questionnaire, and of these, 30 (88 %) completed the follow-up interview. Finally, we were able to locate medical records for 26 (87 %) of the women that completed the follow-up interview.

In the expanded sample, there were 763 additional UCRM patients first seen in 2004, of whom 175 responded that they were interested in participating. Ninety-two (53 %) of these women screened eligible. Of these women, 64 (68 %) completed both components of the *FEQ*. Of these, we were able to locate 41 (64 %) medical records for review. Subsequently, we determined that four of these women were seen at UCRM prior to 2004 and we removed them from the final analysis. Thus, the total sample included in the validation analysis is 63 women: 26 from the initial sample, plus 37 from the expanded sample.

The characteristics of the women enrolled are shown in Table 2, with comparison to all patients seen for first fertility visits at the UCRM in 2004 (where available from billing data). In general, participants were highly educated and with medium to high household income. About 30 % had never had a pregnancy. Participants were slightly younger than clinic patients, with a mean age of 30.4 years, compared to the clinic population mean of 31.8 years (p = 0.066). Participants were probably more likely to have had a successful pregnancy, as 65.0 % of participants had a subsequent visit to evaluate an early pregnancy by ultrasound, whereas 49.0 % of all clinic patients had such an evaluation (p = 0.014).

**Table 2:** Characteristics of the women included in the validation study and of their source population

	Women in validation study (*n* = 63)	All other patients^a^ (*N* = 860)	P value^**^
	Mean (S.D.)	Mean (S.D.)	
Years of age	30.4 (4.7)	31.8 (5.8)	0.066
Visit type	N (%)	N (%)	
Initial infertility visit	48 (76.2)	739 (85.9)	0.035
Initial IVF visit	15 (23.8)	121 (14.1)	
Had early pregnancy ultrasound at UCRM	41 (65.0)	422 (49.0)	0.014
Race			
White	45 (67.2)	NA	
Other	8 (11.9)		
Household income (in US dollars)			
12,000 - 25,000	2 (3.0)	NA	
25,001 - 50,000	10 (14.9)		
50,001 - 75,000	14 (20.9)		
75,001 - 100,000	25 (39.7)		
Over 100,000	11 (16.4)		
Education			
Graduated high school	3 (4.8)	NA	
Some college/vocational school	11 (17.6)		
Graduated college	28 (44.4)		
Attended graduate school	21 (33.3)		
Outcome of fertility treatment			
no pregnancy	20 (29.9)	NA	
pregnancy, no live birth	4 (6.0)		
live birth	42 (62.7)		

Percentages for some variables do not add to 100 % because of missing data

*NA* not available

^**^Comparing women in validation study to all other female patients seen for infertility at the Utah Center for Reproductive Medicine, with the first visit in 2004

^a^All other new female patients seen for infertility at the Utah Center for Reproductive Medicine, with the first visit in 2004

### Treatment history

The agreement between the *FEQ* and the medical record for different treatments is shown in Table 3. Compared to the medical record, the sensitivity of the *FEQ* was uniformly higher than specificity. The agreement was good for intrauterine insemination and assisted reproductive technology (kappa 0.64, 95%CI 0.46-0.83; and 0.74, 95%CI 0.57-0.90, respectively), but lower for use of oral ovulation drugs and injectable ovulation drugs (0.41, 95%CI 0.21-0.61 and 0.21,95%CI 0.0.-0.51, respectively). For women who never conceived (*n* = 29), the respective kappas were very similar: 0.65 for intrauterine insemination, 0.68 for assisted reproductive technology , 0.45 for oral ovulation drugs, and 0.22 for injective ovulation drugs, respectively.

**Table 3:** Agreement between medical record review and *FEQ* interview for fertility treatments, and sensitivity and specificity of the *FEQ* interview, considering the medical record as the gold standard^a^

				Cohen’s		
Ovulation drugs, oral				Kappa [95 % CI]	Sensitivity [95 % CI]	Specificity [95 % CI]
		Interview				
		Yes	No	0.41 [0.23-0.93]	91 % [80, 100]	55 % [39, 71]
Medical record	Yes	21	2			
	No	17	21			
Ovulation drugs, injectable						
		Interview				
		Yes	No	0.26 [0.03-0.51]	70 % [54, 85]	57 % [39, 75]
Medical record	Yes	23	10			
	No	12	16			
Intrauterine insemination						
		Interview				
		Yes	No	0.64 [0.46-0.83]	93 % [83, 100]	69 % [59, 88]
Medical record	Yes	25	2			
	No	9	25			
In vitro fertilization						
		Interview				
		Yes	No	0.74 [0.57-0.90]	96 % [88, 100]	82 % [69, 94]
Medical record	Yes	23	1			
	No	7	30			
Pregnancy						
		Interview				
		Yes	No	0.65 [0.47-0.84]	97 % [91, 100]	67 % [49, 84]
Medical record	Yes	32	1			
	No	9	18			
Live birth						
		Interview				
		Yes	No	0.55 [0.36-0.75]	96 % [87, 100]	64 % [47, 80]
Medical record	Yes	22	1			
	No	12	21			

^a^Some items were missing in the medical record for some patients, so actual number for each item is less than 63

### Pregnancy history

The kappa for the agreement for pregnancy history during the time the woman was a patient at UCRM was 0.65. There was perfect concordance for 50 (79.4 %) participants with respect to the number of pregnancies reported in the interview with the number of pregnancies reported in the medical record. Nine women (14.3 %) reported more pregnancies in the interview than were reported in the medical record, while one (1.6 %) reported one fewer pregnancy in the interview than was reported in the medical record. The kappa for the agreement for live births a woman had during the time she was a patient at UCRM was 0.55. 43 (68.3 %) showed perfect concordance, while 12 (19 %) reported more live births in the interview than in the medical record and one (1.6 %) women reported one fewer live birth in the interview than in the medical record.

### Time at risk for pregnancy and to time to pregnancy

**Fig. 2:**
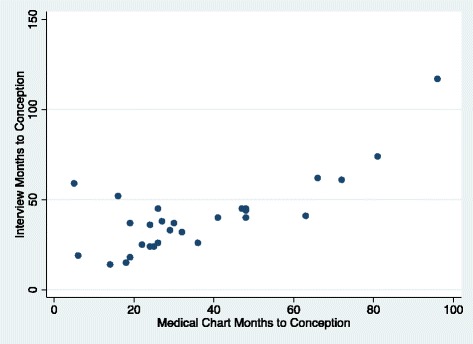
Scatter Plot of time to pregnancy as reported in medical record compared to self-report from interview

About half of the medical records did not contain sufficiently detailed information on time attempting to conceive at the first visit. We were able to compare and calculate time at risk for pregnancy for 35 women, and time to pregnancy for 29 of those women. The mean and median time at risk for pregnancy, as reported in the interviews was 42.1 months and 40 months respectively; in the medical record it was 36.4 and 30, respectively, with a Pearson’s correlation coefficient of 0.42 (95 % CI: 0.10-0.66) (Table 4). For time to pregnancy, the Pearson’s correlation coefficient was 0.77 (95 % CI: 0.55-0.88) (Table 4 and Fig. 2).

**Table 4:** Correlation between *FEQ* interview and medical record review for duration of time at risk for pregnancy and time to pregnancy

	Source	Mean Months (SD)	Median Months	Range, Months	Correlation^a^ [95 % CI]
Time at risk for pregnancy, *n* = 35	Interview	42.1 (22.5)	40	7–117	0.42 [0.10-0.66]
	Medical record	36.4 (21.7)	30	5 –96	
Time to pregnancy, *n* = 29	Interview	40.5 (20.8)	38	14–117	0.77 [0.55-0.88]
	Medical record	36.4 (22.3)	29	5–96	

^a^Pearson’s Rho

## Discussion

We designed the *FEQ* to cover time at risk of pregnancy, fertility treatments received, pregnancy outcomes from those treatments, and a wide range of factors that may influence choices about fertility treatments. In this initial validation study, we found that women’s responses to the *FEQ* were reasonably comparable to medical records for total time at risk for pregnancy, time to pregnancy, pregnancy, live birth, and the use of IVF and artificial insemination. However, there was poor correlation between the *FEQ* and medical records for the use of oral or injectable ovulation drugs. Uniformly, sensitivity was higher than specificity, meaning that women reported many treatments or events that were not found in the medical record. We believe it is likely that some women may have obtained treatments from physicians outside of the UCRM (since we did not have medical record information from other clinics and in the *FEQ* did not ask women at which clinic they had received each treatment). Alternatively, it is possible that women misunderstood the questions, or that the use of some treatments was not completely recorded in the medical record. Underreporting in the medical record of treatments actually given at the UCRM is possible for drugs, but we believe it is much less likely for procedures such as artificial insemination or IVF.

Although there was substantial agreement in regards to pregnancies a woman achieved while as a patient in the clinic, 9 (14 %) of the women interviewed reported having more pregnancies at the time they were patients at UCRM than those recorded in their medical record. This is consistent with the fact that after receiving fertility treatment at the UCRM, some women go to their own obstetricians, family physicians, or midwives to confirm a pregnancy and receive prenatal care (since prenatal care is not provided at the UCRM). Unless there is some subsequent contact between the women and the UCRM, these pregnancies are not documented in the UCRM records. During this time period, there was routine follow-up from UCRM to women or couples undergoing IVF, but not necessarily for women undergoing other types of fertility treatment. Probably for the same reasons, many women who had live births reported more live births in their interviews than the number found in their UCRM medical record. Additional validation studies that allow linkage to medical records of *all* providers seen, or perhaps complete visit and pharmacy billing records, are needed to corroborate our hypothesis that women reported additional fertility treatment and care for pregnancy outside of the single specialty clinic studied.

We believe that the most unique and innovative aspect of the *FEQ* is that it captures a more accurate and complete time at risk for pregnancy (called “attempts to conceive” in the *FEQ*) than the single measure of time “trying to conceive” reported in the woman’s medical record, as evidenced by the 35 (52 %) women in our sample that reported multiple attempts in the time leading up to the first clinic visit (for which the clinic visit identified only one “time trying” to conceive). A woman might report a longer duration of trying to conceive during her first clinic visit than exists in reality, either because she is anxious to start treatment or because she did not take into account any gaps between her attempts to conceive (such as miscarriages, times of separation, or temporary use of birth control). On the other hand, a woman may also report a shorter duration than her actual time attempting to conceive. This may occur when a woman started having sexual intercourse without contraception thereby putting herself at risk of pregnancy but did not consider herself to be “trying” to achieve pregnancy until a later point of time. For these reasons, we have reported the correlation between the *FEQ* interview and the medical record for time at risk for pregnancy, but did not consider the medical record as a “gold standard” for this information.

In the development and pilot testing of the *FEQ*, we found that a combination of written or online questions, followed by a clarifying telephone interview seemed to assure adequate understanding of the concept of “attempt to conceive.” We found that the written description of “attempt to conceive” (as found in the full *FEQ* shown in the Additional file 1) was often not sufficiently understood in the written questionnaire alone. This was the key issue that led us to develop the *FEQ* in a mixed mode, two-step format, with the interview component focused on clarifying and expanding the information specifically tied to each attempt to conceive. While this makes the *FEQ* more resource-intensive to administer than an online-only (or written-only) questionnaire, we anticipate that for some research aims and settings, it may be worthwhile to collect this detailed information on time at risk for pregnancy. Other investigators have also used interviews to assess information about time at risk of pregnancy across different pregnancies and attempts [23]. While time at risk for pregnancy assessed retrospectively may be subject to recall bias, others have shown that the reliability of time to pregnancy recall is enhanced when women are queried in-person or via telephone or e-mail [21, 25, 26]. We believe this supports the value of the two-stage approach to assessing time at risk for pregnancy we have employed in the *FEQ*. However, for other research aims and settings, the written (online or paper) questionnaire alone may suffice. We plan future research to assess the two-stage, mixed mode assessment in a prospective cohort.

The *FEQ* also contains many additional elements not validated in this study, although many of them have been used, and some validated, in other studies (see Table 1). Not all of these elements may be pertinent for other research uses where the *FEQ* may be useful.

Women who participated in this study were not completely reflective of all patients seen at the UCRM in 2004. Among the original sample, 30 of 69 (43 %) women contacted ultimately completed both components of *FEQ*; while in the expanded sample 68 % of those eligible completed both components. The difference in response rate may be related to the $10 compensation provided to the latter group. Those that participated in this validation study were probably more likely to have conceived while under care at UCRM, as we infer from the higher proportion of participants who received visits for early ultrasound assessment of pregnancy (Table 2). Women who had experienced success were likely more motivated to participate in the study. We cannot exclude the possibility that recall of events may be less complete for women who did not conceive with treatment. There was limited socioeconomic and ethnic diversity in the clinic population and the study sample. It would be useful to focus further validation studies on women from broader cultural and socioeconomic backgrounds, and to include women who did not conceive with treatment.

## Conclusions

This study provides initial validation for key elements elements of a mixed mode questionnaire that can be used to improve our understanding of the epidemiology of subfertility in both clinical and population settings, with focus on the natural history of time at risk at pregnancy, and its relationship to fertility treatments and pregnancy outcomes. Our results show high sensitivity for medical treatments, pregnancies, and live births. As suggested by other investigators, we have parsed the time aspect for each attempt to conceive, any fertility treatments received, and any pregnancy each woman may have had in the construction of the questionnaire so that misclassification and bias is minimized to the extent feasible in a retrospective questionnaire [27]. We encourage additional validation from prospective studies. Such information can help inform efforts to better to serve the needs of subfertile couples in the clinical and public health setting.

## Additional file

Additional file 1:
**Fertility experiences woman’s questionnaire.**

## References

[1] Inhorn MC (2003). Global infertility and the globalization of new reproductive technologies: illustrations from Egypt. Soc Sci Med.

[2] Juul S, Karmaus W, Olsen J (1999). Regional differences in waiting time to pregnancy: pregnancy-based surveys from Denmark, France, Germany, Italy and Sweden. The European Infertility and Subfecundity Study Group. Hum Reprod.

[3] Thoma ME, McLain AC, Louis JF, King RB, Trumble AC, Sundaram R, Buck Louis GM (2013). Prevalence of infertility in the United States as estimated by the current duration approach and a traditional constructed approach. Fertil Steril.

[4] Slama R, Ducot B, Carstensen L, Lorente C, de La Rochebrochard E, Leridon H, Keiding N, Bouyer J (2006). Feasibility of the current-duration approach to studying human fecundity. Epidemiology.

[5] Chandra A, Martinez GM, Mosher WD, Abma JC, Jones J (2005). Fertility, family planning, and reproductive health of U.S. women: data from the 2002 National Survey of Family Growth. Vital and Health Statistics Series 23: Data from the National Survey of Family Growth.

[6] Oakley L, Doyle P, Maconochie N (2008). Lifetime prevalence of infertility and infertility treatment in the UK: results from a population-based survey of reproduction. Hum Reprod.

[7] Boivin J, Bunting L, Collins JA, Nygren KG (2007). International estimates of infertility prevalence and treatment-seeking: potential need and demand for infertility medical care. Hum Reprod.

[8] Kessler LM, Craig BM, Plosker SM, Reed DR, Quinn GP (2013). Infertility evaluation and treatment among women in the United States. Fertil Steril.

[9] Hammoud AO, Gibson M, Stanford J, White G, Carrell DT, Peterson M (2009). In vitro fertilization availability and utilization in the United States: a study of demographic, social, and economic factors. Fertil Steril.

[10] Herbert DL, Lucke JC, Dobson AJ (2009). Infertility, medical advice and treatment with fertility hormones and/or in vitro fertilisation: a population perspective from the Australian Longitudinal Study on Women's Health. Aust N Z J Public Health.

[11] Maconochie N, Doyle P, Prior S (2004). The National Women's Health Study: assembly and description of a population-based reproductive cohort. BMC Public Health.

[12] Schmidt L, Holstein BE, Boivin J, Sangren H, Tjornhoj-Thomsen T, Blaabjerg J, Hald F, Andersen AN, Rasmussen PE (2003). Patients' attitudes to medical and psychosocial aspects of care in fertility clinics: findings from the Copenhagen Multi-centre Psychosocial Infertility (COMPI) Research Programme. Hum Reprod.

[13] Camarano L, Alkon A, Nachtigall RD, Schembri M, Weiss S, Croughan MS (2012). Preterm delivery and low birth weight in singleton pregnancies conceived by women with and without a history of infertility. Fertil Steril.

[14] Slade P, O'Neill C, Simpson AJ, Lashen H (2007). The relationship between perceived stigma, disclosure patterns, support and distress in new attendees at an infertility clinic. Hum Reprod.

[15] Newton CR, Sherrard W, Glavac I (1999). The fertility problem inventory: measuring perceived infertility-related stress. Fertil Steril.

[16] Klonoff-Cohen H, Natarajan L (2004). The concerns during assisted reproductive technologies (CART) scale and pregnancy outcomes. Fertil Steril.

[17] Hjelmstedt A, Andersson L, Skoog-Svanberg A, Bergh T, Boivin J, Collins A (1999). Gender differences in psychological reactions to infertility among couples seeking IVF- and ICSI-treatment. Acta Obstet Gynecol Scand.

[18] Moreau C, Bouyer J, Ducot B, Spira A, Slama R (2008). When do involuntarily infertile couples choose to seek medical help?. Fertil Steril.

[19] White L, McQuillan J, Greil AL (2006). Explaining disparities in treatment seeking: the case of infertility. Fertil Steril.

[20] Oddens BJ, den Tonkelaar I, Nieuwenhuyse H (1999). Psychosocial experiences in women facing fertility problems--a comparative survey. Hum Reprod.

[21] Cooney MA, Buck Louis GM, Sundaram R, McGuiness BM, Lynch CD (2009). Validity of self-reported time to pregnancy. Epidemiology.

[22] Greil AL, McQuillan J, Johnson K, Slauson-Blevins K, Shreffler KM (2010). The hidden infertile: infertile women without pregnancy intent in the United States. Fertil Steril.

[23] Juul S, Karmaus W, Olsen J, The European Infertility and Subfecundity Study Group (1999). Regional differences in waiting time to pregnancy: pregnancy-based surveys from Denmark, France, Germany. Italy Sweden Hum Reprod.

[24] Viera AJ, Garrett JM (2005). Understanding interobserver agreement: the kappa statistic. Fam Med.

[25] Zielhuis GA, Hulscher ME, Florack EI (1992). Validity and reliability of a questionnaire on fecundability. Int J Epidemiol.

[26] Joffe M, Villard L, Li Z, Plowman R, Vessey M (1995). A time to pregnancy questionnaire designed for long term recall: validity in Oxford. England J Epidemiol Community Health.

[27] Hvidtjorn D, Grove J, Schendel D, Schieve LA, Ernst E, Olsen J, Thorsen P (2009). Validation of self-reported data on assisted conception in The Danish National Birth Cohort. Hum Reprod.

[28] Stone MB, Stanford JB, Lyon JL, Vanderslice JA, Alder SC (2012). Childhood thyroid radioiodine exposure and subsequent infertility in the Intermountain Fallout Cohort. Environ Health Perspect.

[29] Mosher WD, Jones J, Abma JC (2012). Intended and unintended births in the United States: 1982–2010. Natl Health Stat Report.

