# Supplementary material for: Development and initial validation of a fertility experiences questionnaire
Source: Reprod Health. 2015 Jul 17;12:62. doi: 10.1186/s12978-015-0054-3 (PMC4504457; doi:10.1186/s12978-015-0054-3)
Supplement: Additional file 1: — Fertility experiences woman’s questionnaire. [file 12978_2015_54_MOESM1_ESM.pdf]

Fertility Experiences
Woman's Questionnaire

This file includes the written (online or paper) portion of the Fertility Experiences Questionnaire (pages 1-18), followed by the telephone interview portion of the questionnaire (pages 19-29). Those items that were assessed independently in the medical record review are highlighted and appear on pages 5 and 6 of the written portion and pages 2,3,5,6,7 of the telephone interview (pages 20,21,23,24,25 of the overall document).

The Purpose of the Study of Fertility Experiences

Thank you for your participation in this study! We appreciate your willingness to participate in this important research. This questionnaire is part of a study to assess the experiences of couples about getting pregnant or not getting pregnant. We are studying whether fertility treatments were used and important outcomes of any pregnancies that happened.

Online version only] This questionnaire is administered with secure computing standards to ensure your privacy. Only authorized study personnel will have access to your responses. All answers are completely confidential.

This study is being conducted through the Department of Family and Preventive Medicine at the University of Utah, Salt Lake City, Utah. The Principal Investigator is Dr. Joseph Stanford. He can be reached at 801-587-3331 or joseph.stanford@utah.edu. If you have questions regarding your rights as a research participant, or if problems arise which you do not feel you can discuss with the Investigator, please contact the Institutional Review Board Office at the University of Utah, at 801-581-3655.

Instructions for Completing the Questionnaire

Please take your time as you answer the following questions. Be as accurate as you can, but feel free to make your best guess if you are unsure of the exact answer.

[Paper version only] Please send your completed questionnaire to [name] at [name]@utah.edu, you can fax it to [fax number], or you can mail it to [name], Division of Public Health, University of Utah, 375 Chipeta Way Suite A, Salt Lake City, UT 84108. If you have any questions or comments please talk to [name], Study Coordinator.

[Online version only] When you are done with a set of questions on the screen, click on the "Next" button to continue. If you want to go back to a previous screen and change your answers, click the "Previous" button. If you want to save your responses and come back later to finish the questionnaire, please click the "Save and Return Later" button. When you click this button, please type in your email so that a return ticket may be sent to you. The return

ticket is a code that will allow you to retrieve your incomplete questionnaire and finish it. Once you complete the questionnaire, please click the "Submit" button.

Fertility Experiences
Woman's Questionnaire

Study ID# ____________________________

Initials of your first and last name: ________

Today's Date |___|___|___| / |___|___| / ___|___|___|___| (*example:* Mar / 17 / 2005)
Month / Day / Year

A. General Health

(A-01) How tall are you? |___ft|___in| (*example:* 5 ft/ 3 in)

(A-02) How much do you weigh? _________ lbs (*example:* 118 lbs)

(A-03) On how many of the past 7 days did you exercise or participate in sports activities for at least 20 minutes that made you SWEAT and BREATHE HARD, such as fast walking, jogging, swimming laps, playing tennis, fast bicycling, heavy yard work or housework, or similar aerobic activities? Please mark ☒ one.
☐ 0 ☐ 1 ☐ 2 ☐ 3 ☐ 4 ☐ 5 ☐ 6 ☐ 7 ☐ Prefer not to answer

(A-04) On how many of the past 7 days did you exercise or participate in sports activities for at least 20 minutes but less vigorously than described above? Please mark ☒ one.
☐ 0 ☐ 1 ☐ 2 ☐ 3 ☐ 4 ☐ 5 ☐ 6 ☐ 7 ☐ Prefer not to answer

(A-05) In a typical week, how many days on average do you exercise or participate in sports activities for at least 20 minutes that made you SWEAT and BREATHE HARD, such as fast walking, jogging, swimming laps, playing tennis, fast bicycling, heavy yard work or housework, or similar aerobic activities? Please mark ☒ one.
☐ 0 ☐ 1 ☐ 2 ☐ 3 ☐ 4 ☐ 5 ☐ 6 ☐ 7 ☐ Prefer not to answer

(A-06) In a typical week, how many days on average do you exercise or participate in sports activities for at least 20 minutes but less vigorously than described above? Please mark ☒ one.
☐ 0 ☐ 1 ☐ 2 ☐ 3 ☐ 4 ☐ 5 ☐ 6 ☐ 7 ☐ Prefer not to answer

(A-07) Have you smoked at least 100 cigarettes in your entire life (100 cigarettes = 5 packs)?
☐ Yes ☐ No ☐ Don't Know/Not Sure ☐ Prefer not to answer
→If **no**, please skip to question A-11 below

(A-08) Do you now smoke cigarettes every day, some days, or not at all?
☐ Every Day ☐ Some Days ☐ Not at all ☐ Don't Know/Not Sure ☐ Prefer not to answer

(A-09) During the past 12 months, have you stopped smoking for one day or longer because you were trying to quit smoking?
☐ Yes ☐ No ☐ Don't Know/Not Sure ☐ Prefer not to answer

(A-10) How long has it been since you last smoked cigarettes regularly?
☐ Within the past month (less than 1 month ago)
☐ Within the past 3 months (1 month but less than 3 months ago)
☐ Within the past 6 months (3 months but less than 6 months ago)
☐ Within the past year (6 months but less than 1 year ago)
☐ Within the past 5 years (1 year but less than 5 years ago)
☐ Within the past 10 years (5 years but less than 10 years ago)
☐ 10 years or more

☐ Never smoked regularly
☐ Don't Know/Not Sure
☐ Prefer not to answer

(A-11) Do you currently use chewing tobacco, snuff, or snus every day, some days, or not at all?
☐ Every Day ☐ Some Days ☐ Not at all ☐ Don't Know/Not Sure
☐ Prefer not to answer

(A-12) On average during the last month, how many cups of coffee did you drink per **day**?
Do not count espresso because I will ask you about that separately. Please mark ☒ one.
☐ 0 ☐ less than 1 ☐ 1 ☐ 2 ☐ 3 ☐ 4 ☐ 5 ☐ 6 ☐ 7 or more
☐ Prefer not to answer

(A-13) On average during the last month, how many cups of espresso did you drink per **day**? Please mark ☒ one.
☐ 0 ☐ less than 1 ☐ 1 ☐ 2 ☐ 3 ☐ 4 ☐ 5 ☐ 6 ☐ 7 or more
☐ Prefer not to answer

(A-14) On average during the last month, how many cans or bottles of caffeinated soda drinks did you drink per **day**, including Coca Cola, Pepsi, and others? Please mark ☒ one.
☐ 0 ☐ less than 1 ☐ 1 ☐ 2 ☐ 3 ☐ 4 ☐ 5 ☐ 6 ☐ 7 or more ☐
Prefer not to answer

(A-15) On average, how many units of alcohol do you drink per **week**? (1 unit = glass (half-pint) of beer, 1 measure of spirits, 1 small glass of wine) Please mark ☒ one.
☐ 0 ☐ 1-2 ☐ 3-4 ☐ 5-7 ☐ 8-9 ☐ 10-12 ☐ 13-15 ☐ over 15
☐ Prefer not to answer

(A-16) In the last month, what is the highest number of units of alcohol you had in a **24-hour period**? Please mark ☒ one.
☐ 0 ☐ 1-2 ☐ 3-4 ☐ 5-7 ☐ 8-9 ☐ 10-12 ☐ 13-15 ☐ over 15 ☐
Prefer not to answer

(A-17) Do you have or have you ever had (check all that apply). Please check "None" if you have not had any of these conditions.:
☐ Adult acne ☐ Diabetes ☐ Lung disease (asthma, bronchitis, pneumonia)
☐ Anemia ☐ Pelvic pain ☐ Painful periods (dysmenorrhea)
☐ Pain with intercourse ☐ Appendicitis ☐ Gallbladder disease
☐ Rheumatoid Arthritis ☐ Hair loss ☐ Seizures
☐ Heat/cold intolerance ☐ Thyroid disease ☐ Heart disease
☐ Hepatitis ☐ Breast (nipple) discharge
☐ Excess hair growth (hirsutism) ☐ Breast disease ☐ High blood pressure
☐ Hot flashes ☐ Kidney disease ☐ Liver disease
☐ German Measles (rubella) ☐ Cancer ☐ Colitis
☐ Cystic fibrosis ☐ Neurological disease ☐
Color blindness ☐ Poor sense of smell ☐ Major depression
☐ Anxiety disorder ☐ Bipolar disorder ☐ Blood clots in veins
☐ Chronic Fatigue Syndrome/Fibromyalgia ☐ Ulcerative colitis
☐ Crohn's Disease ☐ Siogren's Syndrome ☐ Scleroderma
☐ Insulin-dependent diabetes mellitus ☐ Non-insulin dependent diabetes mellitus
☐ Food intolerance ☐ Celiac Disease ☐ None
☐Prefer not to answer

(A-18) When was your last Pap smear? |___|___|___| / |___|___|___|___|
☐ Prefer not to answer **Month** / **Year**
(*example:* Mar / 1985)

(A-19) What kind of provider did you get the pap smear from?
☐ Family Physician ☐ Ob/Gyn ☐ Nurse practitioner/physician assistant
☐ Other ☐ Don't know ☐ Prefer not to answer

B. Menstrual History

For this section, please describe the menstrual periods you have had when not taking any hormones (birth control pills or Provera) or any fertility treatments (Clomid or fertility shots).

(B-01) At what age did you have your first menstrual period? _____________ (Age)
☐ Prefer not to answer

(B-02) Your menstrual period almost always comes about monthly.
☐ Yes ☐ No ☐ Prefer not to answer

(B-03) Your menstrual period is usually the same length and heaviness each time.
☐ Yes ☐ No ☐ Prefer not to answer

(B-04) Your menstrual period lasts at least three days.
☐ Yes ☐ No ☐ Prefer not to answer

→If **yes** to Questions B-02, B-03, and B-04, please skip to Section C

(B-05) What is the usual number of periods you might expect in one year? (Please mark ☒ one)
☐ 0-1 ☐ 2-4 ☐ 5-7 ☐ 8-10 ☐ Other, please specify ________(Number)
☐ Prefer not to answer

(B-06) Your period flow is: (Please mark ☒ one)
☐ Often different one period to the next ☐ Very light ☐ About normal ☐ Very heavy ☐ Other
☐ Prefer not to answer

(B-07) The length of your period is: (Please mark ☒ one)
☐ Often different one period to the next ☐ <5 days ☐ 5-10 days ☐ >10 days ☐ Other
☐ Prefer not to answer

C. Sexual History

The next few questions will ask about items of your sexual health history that can have an impact on your fertility.

(C-01) How many sexual partners have you had over the course of your lifetime? _______________ (Number)
☐ Prefer not to answer

(C-02) Have you ever been diagnosed with any of the following?

Chlamydia	☐ Yes	☐ No	☐ Unsure
Gonorrhea	☐ Yes	☐ No	☐ Unsure
Pelvic inflammatory disease	☐ Yes	☐ No	☐ Unsure
Any other sexually transmitted infection	☐ Yes	☐ No	☐ Unsure

☐ Prefer not to answer

D. Pregnancies and "Attempts" to Conceive

The next set of questions will ask about your pregnancies and all "attempts" to conceive, whether or not these attempts actually resulted in a pregnancy.

For the purpose of the rest of the questionnaire, "attempt" to conceive means any period of time lasting more than one month when you were having sexual intercourse with a male partner without using any method to prevent pregnancy. We will call that an "attempt" to conceive even if you were not doing anything else to try to get pregnant.

Each "attempt" to conceive could start in one of three ways:

1) **you began to have sexual intercourse and did not use any method to prevent pregnancy**
2) **you were already having sexual intercourse, and you stopped using any method to prevent pregnancy**
3) **you started having sex again after a pregnancy ended and were not using any method to prevent pregnancy**

Each "attempt" to conceive could end in one of three ways

1) **you became pregnant**
2) **you started using any method to prevent pregnancy**
3) **you stopped having sexual intercourse with your partner at that time**

Here is an example:
First attempt:

- **Woman gets married in January 2000 and starts using birth control pills until January 2001**
- **Attempt BEGINS January 2001 when women stops using birth control**
- **Attempt ENDS in September 2001 when her husband was called to active military duty**

Second attempt:

- **Attempt BEGINS when her husband returns from active duty September 2002 and woman is not on birth control**
- **Because she was not getting pregnant, she saw a doctor and got medication in March 2004**
- **Attempt ENDS in September 2004 when she got pregnant**

Third attempt:

- **Woman had miscarriage in October 2004 and couple decide to use condoms for a few months**
- **Attempt BEGINS in December 2004 when couple stops using condoms**
- **Attempt ENDS in December 2006 when doctor advises woman to use birth control pills for medical reasons**

(D-01) How many times have you ever been pregnant, counting all pregnancies at all times in your life, regardless of the outcome? ________________ (Number)

☐ Prefer not to answer

→If you have never been pregnant, please skip to Question D-05.

(D-02) How many pregnancies resulted from attempts to conceive, as defined above? ________________(Number)

☐ Prefer not to answer

(D-03) How many pregnancies resulted when you were using a method to prevent pregnancy (any method, regardless of how consistently it was used)? ________________ (Number)

☐ Prefer not to answer

(D-04) Did you have any pregnancies that did not fit into any of the above categories?

☐ Yes ☐ No ☐ Prefer not to answer

If yes, please explain: ____

(D-05) In your lifetime, how many different "attempts" to conceive have you had? In other words, how many periods of time in your life did you have intercourse without using any method to prevent pregnancy (whether or not a pregnancy occurred)?

__________________ (Number)
☐ Prefer not to answer

(D-06) These next questions are about **all** your "attempts" to conceive.

				Answer These Columns if "Attempt" Ended in Pregnancy			
	"Attempt" Begin Date (mm/yyyy)	How "attempt" ended:<br>1: Pregnancy<br>2: Started Method to Avoid Pregnancy<br>3: Illness<br>4: Separation or Living Apart<br>5. Divorce<br>6: Abstaining<br>7: Currently Still Trying	"Attempt" End Date (mm/yyyy)	Date Pregnancy Ended (mm/yyyy)<br>If currently still pregnant, please enter 888	Pregnancy Outcome:<br>1: Live Birth<br>2: Miscarriage<br>3: Ectopic Pregnancy<br>4: Stillbirth<br>5: Molar Pregnancy<br>6: Termination<br>7: Currently pregnant<br>8: Other	Name of Baby (if applicabl e)	State Where Birth Occurred (if applicable)
1							
2							
3							
4							
5							
6							
7							
8							
9							
10							
11							
12							
13							
14							
15							

☐ Prefer not to answer

Note: During our telephone interview, we will be asking you some more detailed questions about these attempts.

E. Previous Medical Evaluations

The next few questions will ask you about any previous fertility-related evaluations you may have had.

Question	Answer	Result
(E-01) Have you had an ultrasound of the uterus and ovaries?	☐ Yes ☐ No<br>☐ Unsure	☐ Normal ☐ Abnormal ☐ Unsure<br>☐ Not applicable/did not have this evaluation
(E-02) Have you had an ultrasound scan of the ovaries to look at ovulation (follicle tracking)?	☐ Yes ☐ No<br>☐ Unsure	☐ Normal ☐ Abnormal ☐ Unsure<br>☐ Not applicable/did not have this evaluation
(E-03) Have you had a hysterosalpingogram (x-ray assessment of the uterus and fallopian tubes)?	☐ Yes ☐ No<br>☐ Unsure	☐ Normal ☐ Abnormal ☐ Unsure<br>☐ Not applicable/did not have this evaluation
(E-04) Have you had a hysteroscopy (camera visualization of uterine cavity)?	☐ Yes ☐ No<br>☐ Unsure	☐ Normal ☐ Abnormal ☐ Unsure<br>☐ Not applicable/did not have this evaluation
(E-05) Have you had an endometrial biopsy (small tissue sample is taken from uterus)?	☐ Yes ☐ No<br>☐ Unsure	☐ Normal ☐ Abnormal ☐ Unsure<br>☐ Not applicable/did not have this evaluation

(E-06) Have you had a D&C (scraping of lining of the womb)?	☐ Yes ☐ No ☐ Unsure	☐ Normal ☐ Abnormal ☐ Unsure ☐ Not applicable/did not have this evaluation
(E-07) Have you had a post-coital test (looking at sperm taken from your cervix after intercourse)?	☐ Yes ☐ No ☐ Unsure	☐ Normal ☐ Abnormal ☐ Unsure ☐ Not applicable/did not have this evaluation
(E-08) Have you had day 3 or early cycle blood tests?	☐ Yes ☐ No ☐ Unsure	☐ Normal ☐ Abnormal ☐ Unsure ☐ Not applicable/did not have this evaluation
(E-09) Have you had day 21 or late cycle blood tests (progesterone or ovulation)?	☐ Yes ☐ No ☐ Unsure	☐ Normal ☐ Abnormal ☐ Unsure ☐ Not applicable/did not have this evaluation
(E-10) Have you ever had blood tests for hormone levels related to your fertility?	☐ Yes ☐ No ☐ Unsure	☐ Normal ☐ Abnormal ☐ Unsure ☐ Not applicable/did not have this evaluation
(E-11) Have you had a saliva hormone test related to your fertility?	☐ Yes ☐ No ☐ Unsure	☐ Normal ☐ Abnormal ☐ Unsure ☐ Not applicable/did not have this evaluation

F. Previous Abdominal or Pelvic Surgeries

(F-01) Which of the following surgeries have you had?

Yes	No	Surgery	How many times?	Date(s) of Surgery (mm/yyyy)
		Caesarean Section (C-Section)		
		Cervical cryotherapy or LEEP (freezing or surgery of the cervix to treat an abnormal pap smear)		
		Cautery, or laser treatment for endometriosis		
		Wedge resection or ovarian drilling for polycystic ovaries		
		Laparoscopy (looking in the abdomen or pelvis with a camera)		
		Laparotomy, or major abdominal or pelvic surgery where an incision is made (does not include C-sections)		
		Ovarian cystectomy, or removal of ovarian cyst		
		Myomectomy, or removal of fibroid tumors		
		Polypectomy, or removal of polyps		
		Tubal Ligation ("tubes tied")		
		Tubal Reconstruction (microsurgery)		
		Other Abdominal or Pelvic Surgery, please describe:		

☐ Prefer not to answer

(F-02) Which of the following surgeries has your partner had?

Yes	No	Surgery	Date(s) of Surgery (mm/yyyy)
		Vasectomy	
		Vasectomy reversal	
		Other urologic surgery<br>Please explain:______________________________________	

☐ Prefer not to answer

G. Previous Diagnoses
Have you or your partner ever been told you have or suspect that you might have:

(G-01) Unexplained infertility
☐ Yes ☐ No ☐ Unsure ☐ Prefer not to answer

(G-02) Endometriosis
☐ Yes ☐ No ☐ Unsure ☐ Prefer not to answer

(G-03) Polycystic ovaries (PCOD, PCOS)
☐ Yes ☐ No ☐ Unsure ☐ Prefer not to answer

(G-04) Low progesterone
☐ Yes ☐ No ☐ Unsure ☐ Prefer not to answer

(G-05) Low estrogen
☐ Yes ☐ No ☐ Unsure ☐ Prefer not to answer

(G-06) Not ovulating
☐ Yes ☐ No ☐ Unsure ☐ Prefer not to answer

(G-07) Abnormal ovulation
☐ Yes ☐ No ☐ Unsure ☐ Prefer not to answer

(G-08) Hostile or limited cervical mucus
☐ Yes ☐ No ☐ Unsure ☐ Prefer not to answer

(G-9) Pelvic adhesions or scar tissue
☐ Yes ☐ No ☐ Unsure ☐ Prefer not to answer

(G-10) Blocked or damaged fallopian tubes
☐ Yes ☐ No ☐ Unsure ☐ Prefer not to answer

(G-11) Fibroids in or on the uterus
☐ Yes ☐ No ☐ Unsure ☐ Prefer not to answer

(G-12) Polyps in the uterus
☐ Yes ☐ No ☐ Unsure ☐ Prefer not to answer

(G-13) Luteinized unruptured follicle (LUF)
☐ Yes ☐ No ☐ Unsure ☐ Prefer not to answer

(G-14) Luteal Phase Defect?
☐ Yes ☐ No ☐ Unsure ☐ Prefer not to answer

(G-15) Male factor infertility or sperm abnormality
☐ Yes ☐ No ☐ Unsure ☐ Prefer not to answer

(G-16) Any other health issues that may be related to your fertility?
☐ Yes ☐ No ☐ Prefer not to answer
If **yes**, please specify: ____

H. Previous Fertility Treatment

These next questions are about fertility-enhancing drugs that may be taken by mouth or by injection.

(H-01) Have you ever been advised by a physician or practitioner to try any fertility-enhancing drugs, such as Clomid, clomiphene, perganol, Follistim, or others?
☐ Yes ☐ No ☐ Prefer not to answer

(H-02) Have you ever used any fertility-enhancing drugs?
☐ Yes ☐ No ☐ Prefer not to answer

(H-03) If you answered **yes** to Question H-02: Did you stop treatment when your doctor still recommended continuing it?
☐ Yes ☐ No ☐ Prefer not to answer

(H-04) If you answered **yes** to Question H-03*:* Why did you stop treatment? Please mark ☒ all that apply.
☐ My beliefs ☐ My partner's beliefs ☐ My family's beliefs
☐ Insurance coverage ☐ My job ☐ Distance to clinic too great
☐ Lack of transportation ☐ Felt treatment was not safe enough for me and my future baby
☐ Concern about procedure ☐ Did not feel ready
☐ Wanted to try other forms of treatment
☐ Felt that getting pregnant was impossible
☐ Felt that treatment wasn't working ☐ Did not know where to go for help
☐ Life was too busy
☐ Thought that a pregnancy would happen eventually
☐ There was a change in my relationship to my partner
☐ Got pregnant ☐ Physically drained
☐ Money ☐ Other demands
☐ Don't know ☐ Other, please describe: ___________________________________
☐ Prefer not to answer

(H-05) If you answered **yes** to question H-01 and **yes** to question H-02:
Why did you use fertility-enhancing drugs? Please mark ☒ all that apply.
☐ Treatment was ethically acceptable to me
☐ Treatment was ethically acceptable to my partner
☐ Treatment was ethically acceptable to my family
☐ Insurance covered procedure ☐ My job allowed me flexibility
☐ Clinic was easily accessible ☐ My doctor recommended it
☐ Felt treatment was safe enough to use for me and the baby
☐ Minimal concern about procedure ☐ Felt that the treatment was likely to work
☐ Felt ready ☐ Cost of procedure was not too great for me
☐ Not applicable ☐ Don't know
☐ Other, please describe: ___________
☐ Prefer not to answer

(H-06) If you answered **yes** to question H-01 and **no** to question H-02*:*
Why did you not use any fertility-enhancing drugs? Please mark ☒ all that apply.
☐ My beliefs ☐ My partner's beliefs ☐ My family's beliefs
☐ Insurance coverage ☐ My job ☐ Distance to clinic too great
☐ Lack of transportation ☐ Felt treatment was not safe enough for me and my future baby
☐ Concern about procedure ☐ Did not feel ready
☐ Wanted to try other forms of treatment
☐ Felt that getting pregnant was impossible
☐ Felt that treatment wouldn't work
☐ Did not know where to go for help ☐ Life was so busy, this never became a priority
☐ Thought that a pregnancy would happen eventually
☐ Got pregnant ☐ Physically drained
☐ Money ☐ Other demands
☐ Don't know ☐ Other, please describe: ___________________________________
☐ Prefer not to answer

These next questions are about artificial insemination, either with your partner's sperm or with donor sperm.

(H-07) Have you ever been advised by a physician or practitioner to try artificial insemination?
☐ Yes ☐ No ☐ Prefer not to answer

(H-08) Have you ever attempted artificial insemination?
☐ Yes ☐ No ☐ Prefer not to answer

(H-09) If you answered **yes** to Question H-08: Did you stop treatment when your doctor still recommended it?
☐ Yes ☐ No ☐ Prefer not to answer

(H-10) If you answered **yes** to Question H-09*:* Why did you stop treatment? Please mark ☒ all that apply.
☐ My beliefs ☐ My partner's beliefs ☐ My family's beliefs
☐ Insurance coverage ☐ My job ☐ Distance to clinic too great
☐ Lack of transportation ☐ Felt treatment was not safe enough for me and my future baby
☐ Concern about procedure ☐ Did not feel ready
☐ Wanted to try other forms of treatment
☐ Felt that getting pregnant was impossible
☐ Felt that treatment wasn't working ☐ Did not know where to go for help
☐ Life was too busy
☐ Thought that a pregnancy would happen eventually
☐ There was a change in my relationship to my partner
☐ Got pregnant ☐ Physically drained
☐ Money ☐ Other demands
☐ Don't know ☐ Other, please describe: ____________________________________
☐ Prefer not to answer

(H-12) If you answered **yes** to question H-07 and **yes** to question H-08:
Why did you use artificial insemination? Please mark ☒ all that apply.
☐ Treatment was ethically acceptable to me
☐ Treatment was ethically acceptable to my partner
☐ Treatment was ethically acceptable to my family
☐ Insurance covered procedure ☐ My job allowed me flexibility
☐ Clinic was easily accessible ☐ My doctor recommended it
☐ Felt treatment was safe enough to use for me and the baby
☐ Minimal concern about procedure ☐ Felt that the treatment was likely to work
☐ Felt ready ☐ Cost of procedure was not too great for me
☐ Not applicable ☐ Don't know
☐ Other, please describe: ___________
☐ Prefer not to answer

(H-13) If you answered **yes** to question H-07 and **no** to question H-08*:*
Why did you not use artificial insemination? Please mark ☒ all that apply.
☐ My beliefs ☐ My partner's beliefs ☐ My family's beliefs
☐ Insurance coverage ☐ My job ☐ Distance to clinic too great
☐ Lack of transportation ☐ Felt treatment was not safe enough for me and my future baby
☐ Concern about procedure ☐ Did not feel ready
☐ Wanted to try other forms of treatment
☐ Felt that getting pregnant was impossible
☐ Felt that treatment wouldn't work
☐ Did not know where to go for help ☐ Life was so busy, this never became a priority
☐ Thought that a pregnancy would happen eventually
☐ Got pregnant ☐ Physically drained
☐ Money ☐ Other demands
☐ Don't know ☐ Other, please describe: ____________________________________
☐ Prefer not to answer

These next questions are about in-vitro fertilization (IVF) or similar ART treatments, such as intra-cytoplasmic sperm injection (ICSI), gamete intra-fallopian transfer (GIFT), or zygote intra-fallopian transfer (ZIFT). By ART treatment, we mean any treatment that involves removing the egg from the woman's body and then replacing the egg or embryo back into the body.

(H-14) Have you ever been advised by a physician or practitioner to try IVF, ICSI, or any other ART.
☐ Yes ☐ No ☐ Prefer not to answer

(H-15) Have you ever attempted IVF, ICSI or any other ART.
☐ Yes ☐ No ☐ Prefer not to answer

(H-16) If you answered **yes** to Question H-15: Did you stop treatment when your doctor still recommended continuing it?
☐ Yes ☐ No ☐ Prefer not to answer

(H-17) If you answered **yes** to Question H-16*:* Why did you stop treatment? Please mark ☒ all that apply.
☐ My beliefs ☐ My partner's beliefs ☐ My family's beliefs
☐ Insurance coverage ☐ My job ☐ Distance to clinic too great
☐ Lack of transportation ☐ Felt treatment was not safe enough for me and my future baby
☐ Concern about procedure ☐ Did not feel ready
☐ Wanted to try other forms of treatment
☐ Felt that getting pregnant was impossible
☐ Felt that treatment wasn't working ☐ Did not know where to go for help
☐ Life was too busy
☐ Thought that a pregnancy would happen eventually
☐ There was a change in my relationship to my partner
☐ Got pregnant ☐ Physically drained
☐ Money ☐ Other demands
☐ Don't know ☐ Other, please describe: ___________________________________
☐ Prefer not to answer

(H-18) If you answered **yes** to question H-14 and **yes** to question H-15:
Why did you use IVF, ICSI, or any other ART? Please mark ☒ all that apply.
☐ Treatment was ethically acceptable to me
☐ Treatment was ethically acceptable to my partner
☐ Treatment was ethically acceptable to my family
☐ Insurance covered procedure ☐ My job allowed me flexibility
☐ Clinic was easily accessible ☐ My doctor recommended it
☐ Felt treatment was safe enough to use for me and the baby
☐ Minimal concern about procedure ☐ Felt that the treatment was likely to work
☐ Felt ready ☐ Cost of procedure was not too great for me
☐ Not applicable ☐ Don't know
☐ Other, please describe: __________
☐ Prefer not to answer

(H-19) If you answered **yes** to question H-14 and **no** to question H-15:
Why did you not use any fertility-enhancing drugs? Please mark ☒ all that apply.
☐ My beliefs ☐ My partner's beliefs ☐ My family's beliefs
☐ Insurance coverage ☐ My job ☐ Distance to clinic too great
☐ Lack of transportation ☐ Felt treatment was not safe enough for me and my future baby
☐ Concern about procedure ☐ Did not feel ready
☐ Wanted to try other forms of treatment
☐ Felt that getting pregnant was impossible
☐ Felt that treatment wouldn't work
☐ Did not know where to go for help ☐ Life was so busy, this never became a priority
☐ Thought that a pregnancy would happen eventually
☐ Got pregnant ☐ Physically drained
☐ Money ☐ Other demands

☐ Don't know ☐ Other, please describe: ____________________
☐ Prefer not to answer

These next questions are about alternative fertility treatments. These may include acupuncture, herbal remedies, fertility diets, etc.

(H-20) Have you ever been advised by a physician or practitioner to try alternative fertility treatments (acupuncture, herbal remedies, fertility diets, etc)?
☐ Yes ☐ No ☐ Prefer not to answer

(H-21) Have you ever attempted any alternative fertility treatments?
☐ Yes ☐ No ☐ Prefer not to answer

(H-22) If you answered **yes** to Question H-21: Did you stop treatment when your doctor still recommended continuing it?
☐ Yes ☐ No ☐ Prefer not to answer

(H-23) If you answered **yes** to Question H-22: Why did you stop treatment? Please mark ☒ all that apply.
☐ My beliefs ☐ My partner's beliefs ☐ My family's beliefs
☐ Insurance coverage ☐ My job ☐ Distance to clinic too great
☐ Lack of transportation ☐ Felt treatment was not safe enough for me and my future baby
☐ Concern about procedure ☐ Did not feel ready
☐ Wanted to try other forms of treatment
☐ Felt that getting pregnant was impossible
☐ Felt that treatment wasn't working ☐ Did not know where to go for help
☐ Life was too busy
☐ Thought that a pregnancy would happen eventually
☐ There was a change in my relationship to my partner
☐ Got pregnant ☐ Physically drained
☐ Money ☐ Other demands
☐ Don't know ☐ Other, please describe: ____________________
☐ Prefer not to answer

(H-24) If you answered **yes** to question H-21:
Why did you use alternative fertility treatment? Please mark ☒ all that apply.
☐ Treatment was ethically acceptable to me
☐ Treatment was ethically acceptable to my partner
☐ Treatment was ethically acceptable to my family
☐ Insurance covered procedure ☐ My job allowed me flexibility
☐ Clinic was easily accessible ☐ My doctor recommended it
☐ Felt treatment was safe enough to use for me and the baby
☐ Minimal concern about procedure ☐ Felt that the treatment was likely to work
☐ Felt ready ☐ Cost of procedure was not too great for me
☐ Not applicable ☐ Don't know
☐ Other, please describe: __________
☐ Prefer not to answer

(H-25) If you answered **no** to question H-22:
Why did you not use alternative fertility treatment? Please mark ☒ all that apply.
☐ My beliefs ☐ My partner's beliefs ☐ My family's beliefs
☐ Insurance coverage ☐ My job ☐ Distance to clinic too great
☐ Lack of transportation ☐ Felt treatment was not safe enough for me and my future baby
☐ Concern about procedure ☐ Did not feel ready
☐ Wanted to try other forms of treatment
☐ Felt that getting pregnant was impossible
☐ Felt that treatment wouldn't work
☐ Did not know where to go for help ☐ Life was so busy, this never became a priority
☐ Thought that a pregnancy would happen eventually
☐ Got pregnant ☐ Physically drained
☐ Money ☐ Other demands

☐ Don't know ☐ Other, please describe: ____________________
☐ Prefer not to answer

I. Adoption

(I-01) Have you ever applied for adoption?
☐ Yes ☐ No ☐ Prefer not to answer

(I-02) Do you have any adopted children?
☐ Yes ☐ No ☐ Prefer not to answer

(I-03) If you answered **yes** to (I-01) and **no** to (I-02):
What was the reason you did not adopt children?
____

☐ Prefer not to answer

(I-04) Have you ever had foster children?
☐ Yes ☐ No ☐ Prefer not to answer

(I-05) Do you currently have any foster children?
☐ Yes ☐ No ☐ Prefer not to answer

(I-06) If you answered **yes** to (I-04) and **no** to (I-05):
What was the reason you did not foster children?
____

☐ Prefer not to answer

J. Stress and Social Situation
These next few questions are about how you or your partner's fertility problems and/or treatment have affected you.

(J-01) With reference to you or your partner's fertility problems and/or treatment, do you feel that:
[Please mark ☒ one answer for each line]

	Strongly Agree	Agree	Neutral	Disagree	Strongly Disagree
My life has changed very much	☐	☐	☐	☐	☐
My life has been disrupted as a result	☐	☐	☐	☐	☐
It has been stressful for me to deal with	☐	☐	☐	☐	☐

☐ Prefer not to answer

(J-02) How have you or your partner's fertility problems affected your marriage/partnership?
[Please mark ☒ one answer for each line]

	Strongly Agree	Agree	Neutral	Disagree	Strongly Disagree
Brought us closer together	☐	☐	☐	☐	☐
Strengthened our relationship	☐	☐	☐	☐	☐
Caused crisis in our relationship	☐	☐	☐	☐	☐
Caused thoughts of divorce	☐	☐	☐	☐	☐

☐ Prefer not to answer

(J-03) How much stress has you or your partner's fertility problems placed on the following?
[Please mark ☒ one answer for each line]

	A lot	Some	A little	None
Your marriage/partnership	☐	☐	☐	☐

Your sex life	☐	☐	☐	☐
Your relationships with your family	☐	☐	☐	☐
Your relationships with your family-in-law	☐	☐	☐	☐
Your relationships with friends	☐	☐	☐	☐
Your relationships with workmates	☐	☐	☐	☐
Your relationships to people with children	☐	☐	☐	☐
Your relationships to pregnant women	☐	☐	☐	☐
Your physical health	☐	☐	☐	☐
Your mental health	☐	☐	☐	☐
Your financial condition	☐	☐	☐	☐

☐ Prefer not to answer

(J-04) Have you received support and understanding from any of the following people in relation to you or your partner's fertility problems or treatment? [Please mark ⊠ one answer for each line]

	Always	Often	Sometimes	Rarely	Never	Don't have
Family	☐	☐	☐	☐	☐	☐
Partner	☐	☐	☐	☐	☐	☐
Partner's Family	☐	☐	☐	☐	☐	☐
Friends	☐	☐	☐	☐	☐	☐
Colleagues	☐	☐	☐	☐	☐	☐
Support Group	☐	☐	☐	☐	☐	☐
Therapist/Counselor	☐	☐	☐	☐	☐	☐
Others	☐	☐	☐	☐	☐	☐

Who? ____

☐ Prefer not to answer

(J-05) Have you experienced that some people react negatively to you or your partner's fertility problems or treatment? [Please mark ⊠ one answer for each line]

	Always	Often	Sometimes	Rarely	Never	Don't have
Family	☐	☐	☐	☐	☐	☐
Partner	☐	☐	☐	☐	☐	☐
Partner's Family	☐	☐	☐	☐	☐	☐
Friends	☐	☐	☐	☐	☐	☐
Colleagues	☐	☐	☐	☐	☐	☐
Support Group	☐	☐	☐	☐	☐	☐
Therapist/Counselor	☐	☐	☐	☐	☐	☐
Others	☐	☐	☐	☐	☐	☐

Who? ____

☐ Prefer not to answer

<u>K. Experience of Past Fertility Treatment</u>

(K-01) Have you or your partner ever been evaluated or treated for fertility problems or miscarriage in the past?

☐ Yes ☐ No ☐ Prefer not to answer

→If **<u>no</u>**, please skip to Section L, Demographic Information

In the next set of questions, please consider your overall experience with medical evaluation(s) and treatment(s) for infertility or miscarriage that you and your partner have had in the past. Please answer from your own perspective, not necessarily from your partner's perspective. This information will be kept completely confidential and will not be shared with your doctors.

How do you assess the doctors and the staff that you have worked with?

(K-02) Did they make you feel you had enough time during the consultations? (Please mark ⊠ one)

Bad Excellent

1 2 3 4 5 Don't know/not relevant
☐ ☐ ☐ ☐ ☐ ☐
☐ Prefer not to answer

(K-03) Did they involve you in decisions? (Please mark ☒ one)
Bad Excellent
1 2 3 4 5 Don't know/not relevant
☐ ☐ ☐ ☐ ☐ ☐
☐ Prefer not to answer

(K-04) Did they listen to you? (Please mark ☒ one)
Bad Excellent
1 2 3 4 5 Don't know/not relevant
☐ ☐ ☐ ☐ ☐ ☐
☐ Prefer not to answer

(K-05) Did they explain the purpose of examinations, tests, and treatments? (Please mark ☒ one)
Bad Excellent
1 2 3 4 5 Don't know/not relevant
☐ ☐ ☐ ☐ ☐ ☐
☐ Prefer not to answer

(K-06) Did they tell you what you wanted to know about the causes of infertility and/or miscarriage? (Please mark ☒ one)
Bad Excellent
1 2 3 4 5 Don't know/not relevant
☐ ☐ ☐ ☐ ☐ ☐
☐ Prefer not to answer

(K-07) Did they tell you what you wanted to know about the treatment of infertility and/or miscarriage? (Please mark ☒ one)
Bad Excellent
1 2 3 4 5 Don't know/not relevant
☐ ☐ ☐ ☐ ☐ ☐
☐ Prefer not to answer

(K-08) Did they deal with emotional consequences of your infertility or miscarriage and treatment? (Please mark ☒ one)
Bad Excellent
1 2 3 4 5 Don't know/not relevant
☐ ☐ ☐ ☐ ☐ ☐
☐ Prefer not to answer

(K-09) Did they make a treatment plan adjusted to your special situation? (Please mark ☒ one)
Bad Excellent
1 2 3 4 5 Don't know/not relevant
☐ ☐ ☐ ☐ ☐ ☐
☐ Prefer not to answer

(K-10) What have you liked most about you and your partner's past treatment?

____

____

____
☐ Prefer not to answer

(K-11) What have you liked least about you and your partner's past treatment?

____

____

____

☐ Prefer not to answer

(K-12) What is your overall satisfaction rating for you and your partner's past treatment, rated from 1-10? (Please mark ☒ one)

Not at all satisfied Very Satisfied

☐ 1 ☐ 2 ☐ 3 ☐ 4 ☐ 5 ☐ 6 ☐ 7 ☐ 8 ☐ 9 ☐ 10

☐ Prefer not to answer

L. Demographic Information

(L-01) What is your marital status? (Please mark ☒ one)

☐ Never married ☐ Married ☐ Widow ☐ Divorced ☐ Prefer not to answer

(L-02) In what month and year did you marry? |___|___|___| / |___|___|___|___|

☐ Prefer not to answer **Month** / **Year**

(*example:* Mar / 1985)

(L-03) Is this your first marriage?

☐ Yes ☐ No ☐ Prefer not to answer

(L-04) If you answered **no** to L-03: What date was your first marriage?

|___|___|___| / |___|___|___|___|

☐ Prefer not to answer **Month** / **Year**

(*example:* Mar / 1985)

(L-05) What is the highest level of education you have had? Please mark ☒ one.

☐ Did not complete high school ☐ Graduated from high school

☐ Some college/vocational school ☐ Graduated from college

☐ Attended graduate school

☐ Prefer not to answer

(L-06) What is your race? Please mark ☒ all that apply.

☐ African American ☐ American Indian/Alaskan Native ☐ Asian

☐ Hawaiian Native/Pacific Islander ☐ White

☐ Other, please specify: ______________________________

☐ Prefer not to answer

(L-07) Are you Hispanic/Latino?

☐ Yes ☐ No ☐ Prefer not to answer

(L-08) In what country were you born?

____

(L-09) In what country was your mother born?

____

(L-10) In what country was your father born?

____

(L-11) Do you speak any language fluently other than English?

☐ Yes ☐ No ☐ Prefer not to answer
→If **no**, please skip to question L-06 below

(L-12) Which language(s)?
____
____
____

(L-13) Which language do you speak best?
____

(L-14) Which language do you write best?
____

(L-15) What language is mainly spoken in your home?
____

(L-16) What is your religious preference? Please mark ☒ one.
☐ Catholic ☐ Islamic ☐ Jewish ☐ Latter-day Saint ☐ Orthodox Christian
☐ Other Christian ☐ Unitarian ☐ Hindu ☐ Buddhist
☐ None ☐ Other, please specify: ______________________________
☐ Prefer not to answer

(L-17) About how often do you usually attend religious or worship services? Please mark ☒ one.
☐ More than once per week ☐ Weekly ☐ Monthly ☐ Less than monthly ☐ Never
☐ Prefer not to answer

(L-18) What is your current occupation? Please mark ☒ all that apply
☐ Professional ☐ Technical ☐ Clerical/Sales ☐ Skilled laborer ☐ Unskilled laborer
☐ Homemaker ☐ Student ☐ Educator
☐ Other, please specify: __________________________________
☐ Prefer not to answer

(L-19) Please mark the choice that most closely approximates your yearly total household income. Please mark ☒ one.
☐ Under $12,000 ☐ $12,001-25,000 ☐ $25,001-50,000 ☐ $50,001-75,000
☐ $75,001-100,000 ☐ Over $100,00 ☐ Prefer not to answer

(L-20) Do you keep any written records or summaries of your fertility experiences?
☐ Yes ☐ No ☐ Prefer not to answer

(L-21) Please provide a telephone number where we may reach you. ____

(L-22) Please provide an e-mail address where we may contact you.

(L-23) What days would be best to contact you by phone? Please mark ☒ all that apply
☐ Monday ☐ Tuesday ☐ Wednesday ☐ Thursday
☐ Friday ☐ Saturday ☐ Sunday

(L-24) What times would be best to contact you by phone? Please mark ☒ all that apply
☐ Mornings (9am-12pm) ☐ Afternoons (12pm-5pm) ☐ Evenings (5pm-9pm) ☐ Other

O: Friends and Family with Infertility

(O-01) Do you have any friends and/or family members who have been diagnosed with infertility?
☐ Yes ☐ No
→If **no**, please end survey

(O-02) How many of your friends and/or family members have been diagnosed with infertility? __________ (number)

(O-03) Have any of your friends and/or family members every received treatment for infertility?
☐ Yes ☐ No
→If **no**, please end survey

(O-04) How many of your friends and/or family members have received treatment for infertility? __________ (number)

See below for a list of different treatments for infertility. For each treatment, please mark whether you have a friend of family member who has used the treatment and whether they were successful or unsuccessful at getting pregnant while using the treatment. If more than one family member has used the treatment, you may check the boxes in multiple columns.

Type of Treatment	A friend or family member used this type of treatment to become pregnant	A friend or family member used this type of treatment but did not become pregnant	None of my friends or family members have used this type of treatment	I don't know if my friends of family members have used this type of treatment
Taking their basal body temperature				
Using urine LH test kits (urine ovulation test kits)				
Taking herbs to enhance fertility				
Taking vitamins to enhance fertility				
Used acupuncture to enhance fertility				
Losing weight to enhance fertility				
Adhering to any "fertility" diets				
Monitoring vaginal discharge, cervical mucus, or cervical fluids				
Taking pills to enhance fertility or ovulation, such as clomiphene ("clomid")				
Taking shots to enhance fertility or ovulation				
Artificial insemination				
In vitro fertilization (IVF) with or without ICSI				
Other types of treatment				

Thank you for your participation in this important study!

Telephone interview portion of Fertility Experiences Questionnaire

Conducted after completion of the written portion.

Before starting the interview, review Section D of the written questionnaire, as it pertains to what you will ask her during the interview. Her prior answers from Section D are reproduced below. When you verify these questions, enter the new answer given in the interview, even if it differs from the answer given previously in the written questionnaire.

Some of the questions we discuss today will be duplicating what we asked you on the written portion of the survey. We hope to confirm your answers and add important details. Please bear with us through this process– your answers to these questions are extremely important for the study.

Do you have any questions about the definition of "attempts to conceive" that we are using for this interview?
If she asks how long an attempt to conceive needs to be to qualify, the general guideline is a month or more.
See separate list of special cases that are considered pregnancies and may or may not be considered "attempts to conceive."

I would now like to briefly go over some of the items you wrote down in the questionnaire to ensure accuracy.

(D-01) Counting all pregnancies at all times in your life, regardless of the outcome, you have been pregnant __________________ times. Is this correct?

→*If she has never been pregnant, please skip to "Attempt to Conceive."*

(D-02) _________________ pregnancies resulted from attempts to conceive as previously defined. Is this correct?

(D-03) _________________ pregnancies resulted when you were using a method to prevent pregnancy. This includes any method, regardless of how consistently it was used.

(D-04) You did/did not have any pregnancies that did not fit into any of the above categories.
If yes, please explain: ____

Clarify these and figure out which ones are actually best classified as "attempts to conceive" and which ones are best classified as pregnancies while trying to avoid. See separate list of special cases that are considered pregnancies and may or may not be considered "attempts to conceive." Throughout section M of the interview, refer to the following
(D-06)

				Answer These Columns if "Attempt" Ended in Pregnancy			
	"Attempt" Begin Date (mm/yyyy)	How "attempt" ended:<br>1: Pregnancy<br>2: Started Method to Avoid Pregnancy<br>3: Illness<br>4: Separation or Living Apart<br>5. Divorce<br>6: Abstaining<br>7: Currently Still Trying	"Attempt" End Date (mm/yyyy)	Date Pregnancy Ended (mm/yyyy)<br>If currently still pregnant, please enter 888	Pregnancy Outcome:<br>1: Live Birth<br>2: Miscarriage<br>3: Ectopic Pregnancy<br>4: Stillbirth<br>5: Molar Pregnancy<br>6: Termination<br>7: Currently pregnant<br>8: Other	Name of Baby (if applicabl e)	State Where Birth Occurred (if applicable)
1							
2							
3							
4							
5							

Section M: Attempt to Conceive

I will now ask you a set of questions about each of your “attempts to conceive.”

Attempt # ____________

(M-01) You said you started this attempt in ____________ and ended in ____________, so this attempt lasted for ___________ years and ___________ months. Is this correct? ☐ Still currently attempting to conceive

(M-02) Was this attempt with your *current* partner?
☐ Yes ☐ No ☐ Don’t currently have a partner

(M-03) How did this attempt begin?
☐ you began to have sexual intercourse and did not use any method to prevent pregnancy
☐ you were already having sexual intercourse, and you stopped using any method to prevent pregnancy
☐ you started having sex again after a pregnancy ended and were not using any method to prevent pregnancy
☐ Other:____

(M-04) What was the average frequency of intercourse during this period
Don’t say options aloud; please mark ☒ *one*
☐ <1 time a week ☐ 1-2 times a week ☐ >2 times a week

Feelings and desires about conceiving may change with time. The next question is about your feelings over this particular “attempt to conceive.”

(M-05) On a scale of 0-10, how much did you want to get pregnant during this “attempt to conceive?” 0 means you did not want to conceive, 5 means that you did not care either way if you conceived, and 10 means that you wanted to conceive very much. (*Circle one number*)

0----------1----------2------------3----------4----------5-----------6----------7----------8----------9----------10
Did not want — Did not care either way — Wanted it very much

If your feelings and desires about getting pregnant changed throughout the course of this attempt:
What were they at the beginning of this attempt? ______________________ (Number 0-10)
What were they at the middle of this attempt? ________________________ (Number 0-10)
What were they towards the end of this attempt (or currently, if still attempting?) _____________________ (Number 0-10)

(M-06) On a scale of 0-10, how much do you think your partner wanted you to get pregnant during this “attempt to conceive?” 0 means your partner did not want you to conceive, 5 means that your partner did not care either way if you conceived, and 10 means that your partner wanted you to conceive very much. (*Circle one number*)

0----------1----------2------------3----------4----------5-----------6----------7----------8----------9----------10
Did not want — Did not care either way — Wanted it very much

If your partner’s feelings and desires about getting pregnant changed throughout the course of this attempt:
What were they at the beginning of this attempt? ______________________ (Number 0-10)
What were they at the middle of this attempt? ________________________ (Number 0-10)
What were they towards the end of this attempt (or currently, if still attempting?) _____________________ (Number 0-10)

(M-07) Was there a point during this attempt where you “actively” started trying to get pregnant?
☐ Yes ☐ No
If ***yes***: What date did you actively start trying during this attempt?
|___|___|___| / |___|___|___|___| (*example:* Mar / 1985)
Month / **Year**

(M-08) During this period, did you see a doctor or provider specifically for fertility-related issues? (Please mark ☒ one)
☐ Yes ☐ No ☐ Unsure

→*If **no**, please skip to question M-11.*

(M-09) Did you meet/consult with (*Check **all** that apply*):
- ☐ A general physician/provider (obstetrician, family medicine physician, physician assistant, nurse midwife, or nurse practitioner)?
- ☐ A fertility specialist?
- ☐ An alternative/holistic practitioner (chiropractor, acupuncturist, naturopath, etc.)
- ☐ Other, please describe: ____

(M-10) Did you have health insurance at that time that covered any of the following (check all that apply)?
- ☐ Tests to diagnose fertility problems in you
- ☐ Tests to diagnose fertility problems in your partner
- ☐ Treatment for fertility other than IVF/ART
- ☐ IVF/ART treatment
- ☐ Unsure
- ☐ Other, please describe______________________________

(M-11) How long had you been "attempting to conceive" when you first met with a doctor or provider?
___________years and _____________ months

(M-12) Did this attempt end in a pregnancy (regardless of the pregnancy outcome)?
☐ Yes ☐ No
→ *If **no, skip to M-14***

(M-13) When was the date conception occurred?
Please tell us much as you know.
[Enter as much of the date as they are able to give. This should correspond to the end date for this attempt. Clarify as needed.]

|___|___| /|___|___|___| / |___|___|___|___| (*example:* 12 Mar 1985)
DAY MONTH YEAR
☐ Unsure

The following questions ask about things you may have done to enhance fertility during this attempt to conceive, either on recommendation of a doctor, or on your own.

In order to conceive, have you done any of the following:

**Question**	**During this particular attempt?**	**How much? Insert this column between attempt and month**	**In the month you got pregnant, if there was a pregnancy (mark ☒ if yes)**	**Comments**
(M-14) Timed intercourse by counting the number of days in your menstrual cycle?	☐ Yes ☐ No ☐ Unsure	# cycles/months: _____ or ☐ During the entire attempt ☐ Unsure	☐	
(M-15) Taken your basal body temperature?	☐ Yes ☐ No ☐ Unsure	# cycles/months: _____ or ☐ During the entire attempt	☐	

		☐ Unsure		
(M-16) Used urine LH test kits (urine ovulation test kits)?	☐ Yes ☐ No ☐ Unsure	# cycles/months: ______ or ☐ During the entire attempt ☐ Unsure	☐	
(M-17) Taken herbs intended to enhance fertility?	☐ Yes ☐ No ☐ Unsure	# cycles/months: ______ or ☐ During the entire attempt ☐ Unsure	☐	What kind?
(M-18) Partner taken herbs intended to enhance fertility?	☐ Yes ☐ No ☐ Unsure	# cycles/months: ______ or ☐ During the entire attempt ☐ Unsure	☐	What kind?
(M-19) Taken vitamins intended to enhance fertility?	☐ Yes ☐ No ☐ Unsure	# cycles/months: ______ or ☐ During the entire attempt ☐ Unsure	☐	What kind?
(M-20) Partner taken vitamins intended to enhance fertility?	☐ Yes ☐ No ☐ Unsure	# cycles/months: ______ or ☐ During the entire attempt ☐ Unsure	☐	What kind?
(M-21)Taken any “supplements” to enhance fertility?	☐ Yes ☐ No ☐ Unsure	# cycles/months: ______ or ☐ During the entire attempt ☐ Unsure	☐	What kind?
(M-22) Partner taken any “supplements” to enhance fertility?	☐ Yes ☐ No ☐ Unsure	# cycles/months: ______ or ☐ During the entire attempt ☐ Unsure	☐	What kind?
(M-23) Used acupuncture to enhance fertility?	☐ Yes ☐ No ☐ Unsure	# cycles/months: ______ or ☐ During the entire attempt ☐ Unsure	☐	
(M-24) Partner used acupuncture to enhance fertility?	☐ Yes ☐ No ☐ Unsure	# cycles/months: ______ or ☐ During the entire attempt ☐ Unsure	☐	

(M-25) Lost weight to enhance fertility?	☐ Yes ☐ No ☐ Unsure		☐	Starting wt: ________<br>Ending wt: ________
(M-26) Partner lost weight to enhance fertility?	☐ Yes ☐ No ☐ Unsure		☐	Starting wt: ________<br>Ending wt: ________
(M-27) Adhered to any "fertility" diets?	☐ Yes ☐ No ☐ Unsure	# cycles/months: _____ or ☐ During the entire attempt ☐ Unsure	☐	What kind?
(M-28) Partner adhered to any "fertility" diets?	☐ Yes ☐ No ☐ Unsure	# cycles/months: _____ or ☐ During the entire attempt ☐ Unsure	☐	What kind?
(M-29) Monitored vaginal discharge, cervical mucus, or cervical fluid?	☐ Yes ☐ No ☐ Unsure	# cycles/months: _____ or ☐ During the entire attempt ☐ Unsure	☐	Where did you learn about how to monitor cervical mucus or fluid?
(M-30) Taken any other drug every day to help you ovulate, such as metformin, glucophage, actos, or others?	☐ Yes ☐ No ☐ Unsure	# cycles/months: _____ or ☐ During the entire attempt ☐ Unsure	☐	
(M-31) Taken pills to enhance fertility or ovulation, such as clomiphene ("clomid")?	☐ Yes ☐ No ☐ Unsure	# cycles/months: _____ or ☐ During the entire attempt ☐ Unsure	☐	Which medication?
(M-32) Partner taken pills to enhance fertility such as clomiphene ("clomid")?	☐ Yes ☐ No ☐ Unsure	# cycles/months: _____ or ☐ During the entire attempt ☐ Unsure	☐	Which medication?
(M-33)Taken any hormones to enhance fertility such as progesterone	☐ Yes ☐ No ☐ Unsure	# cycles/months: _____ or ☐ During the entire attempt ☐ Unsure	☐	Which medication?
(M-34) Partner taken any hormones to enhance fertility, energy, or sexuality such as testosterone?	☐ Yes ☐ No ☐ Unsure	# cycles/months: _____ or ☐ During the entire attempt ☐ Unsure	☐	Which medication?
(M-35) Taken shots to enhance fertility or ovulation?	☐ Yes ☐ No ☐ Unsure	# cycles/months: _____ or ☐ During the	☐	Which medication?

		entire attempt<br>☐ Unsure		
(M-36) Had artificial insemination?	☐ Yes ☐ No ☐ Unsure	# cycles/months: ______<br>or<br>☐ During the entire attempt<br>☐ Unsure	☐	
(M-37) Had in vitro fertilization (IVF) with or without ICSI?	☐ Yes ☐ No ☐ Unsure	# cycles/months: ______<br>or<br>☐ During the entire attempt<br>☐ Unsure	☐	
(M-38) Other?	☐ Yes ☐ No ☐ Unsure	# cycles/months: ______<br>or<br>☐ During the entire attempt<br>☐ Unsure	☐	Describe?

(M-39) If patient answered yes to AI or IVF please ask: did you use any donor egg or sperm? ☐ Yes ☐ No

If yes:
(M-40) While doing artificial insemination, did you ever use donor sperm during this attempt? ☐ Yes ☐ No

(M-41) If yes, did you use donor sperm during the cycle that you got pregnant? ☐ Yes ☐ No

(M-42)While doing IVF did you use donor sperm? ☐ Yes ☐ No

(M-43) If yes, did you use donor sperm during the cycle that you got pregnant? ☐ Yes ☐ No

(M-44) While doing IVF did you use donor eggs? ☐ Yes ☐ No

(M-45) If yes, did you use donor eggs during the cycle that you got pregnant? ☐ Yes ☐ No

(M-46) *If she has answered that she has tried ovulation-enhancing drugs, insemination, and/or IVF, please ask:* Can you please expand on the reasons why you chose to pursue (ovulation-enhancing drugs, insemination, and/or IVF)?

____
____
____
____

If this attempt resulted in a pregnancy, skip to M-49

(M-47) How did this attempt end?
☐ Birth Control, Personal ☐ Birth Control, Medical ☐ Divorce/Separation ☐ Illness
☐ Abstaining ☐ Still Attempting

(M-48) When did this attempt end?
|___|___|___| / ___|___|___|___| (*example:* Mar / 2005) ☐ Still currently attempting to conceive
Day Month / Year
→ *Skip to END of Section M.*

(M-49) When did this pregnancy end?
|___|___| /|___|___|___| / |___|___|___|___| (*example:* 12 Mar 1985)
DAY MONTH YEAR
☐ Unsure

(M-50) How far along were you when this pregnancy ended in weeks? (For example 12 weeks gestation, 39 weeks gestation, etc.)
__________________ weeks gestation

(M-51) How many fetuses or babies were in this pregnancy?
Don't read options aloud; please mark ☒ one
☐ One ☐ Twins ☐ Triplets ☐ More than Triplets
☐ Unknown

(M-52) How did this pregnancy end?
Don't read options aloud; please mark ☒ one
☐ Live birth ☐ Miscarriage ☐ Ectopic pregnancy
☐ Stillbirth ☐ Molar pregnancy ☐ Termination
☐ Other, describe: ________________________________
*→If the outcome was **not a live birth**, skip to enter her next "attempt to conceive." If this is her last "attempt to conceive," then skip to Section N. Miscarriage is a pregnancy loss up to 20 weeks; stillbirth is a pregnancy loss after 20 weeks gestation.*

(M-53) What was (were) the baby's(ies') name(s), sex and birth weight?

	First (given name)	Middle	Last (surname)	Birth weight (pounds/ounces or grams)	Sex	Hospital stay of 7 days or more
Baby 1					☐ Male ☐ Female	☐ Yes ☐ No ☐ Unsure
Baby 2					☐ Male ☐ Female	☐ Yes ☐ No ☐ Unsure
Baby 3					☐ Male ☐ Female	☐ Yes ☐ No ☐ Unsure

(M-54) Did you ever breastfed this/these baby(ies)?
☐ Yes ☐ No

(M-55) How long did you breastfed this/these baby(ies)? (in months)
________________ months
*If there were multiple babies with different answers, record the answer for the baby with the **longest** time breastfeeding.*

(M-56) How old was/were this baby(ies) when (s)he first ate something other than breastmilk or water?
________________ months
*If there were multiple babies with different answers, record the answer for the **last** baby to have something besides breastmilk or water.*

END: Recheck to see if all attempt and pregnancy dates are concordant with each other. If she had a pregnancy, review the date the pregnancy ended and how far along she was when the pregnancy ended. If she did not have a pregnancy, review the date the attempt ended. Go over with her again if there is a discrepancy of 2 months or more.
Skip to enter her next "attempt to conceive." If this is her last "attempt to conceive," then skip to Section N.

N. Pregnancies that Happened While You Were Trying to Avoid Pregnancy

Are there any other pregnancies that we did not talk about?
If she has not had any additional pregnancies, please go to section R.
Review and verify the number of pregnancies that happened while trying to avoid pregnancy (from question D-04).

(N-01) Was this pregnancy with your current partner? (Please mark ☒ one)
☐ Yes ☐ No ☐ Don't currently have a partner

Feelings and desires about conceiving may change with time. The next question is about your feelings over this particular event.

(N-02) On a scale of 0-10, how did you feel about getting pregnant during this time? 0 means you did not want to get pregnant, 5 means that you did not care either way if you get pregnant, and 10 means that you wanted very much to get pregnant. (Circle one number)

0----------1----------2-----------3----------4----------5----------6----------7----------8----------9----------10
Did not want Did not care either way Wanted it very much

If your feelings and desires about getting pregnant changed throughout the course of this attempt:
What were they at the beginning of this attempt? ______________________ (Number 0-10)
What were they at the middle of this attempt? ________________________ (Number 0-10)
What were they towards the end of this attempt (or currently, if still attempting?) _____________________ (Number 0-10)

(N-03) On a scale of 0-10, how did you think your partner felt about you getting pregnant during this time? 0 means your partner did not want you to get pregnant, 5 means that your partner did not care either way if you got pregnant, and 10 means that your partner wanted very much for you to get pregnant. (Circle one number)

0----------1----------2-----------3----------4----------5----------6----------7----------8----------9----------10
Did not want Did not care either way Wanted it very much

If your partner's feelings and desires about getting pregnant changed throughout the course of this attempt:
What were they at the beginning of this attempt? ______________________ (Number 0-10)
What were they at the middle of this attempt? ________________________ (Number 0-10)
What were they towards the end of this attempt (or currently, if still attempting?) _____________________ (Number 0-10)

(N-04) In what month and year did this pregnancy end?
|___|___|___| / ___|___|___|___| (*example:* Mar / 2005)
Month / **Year**

(N-05) How far along were you when this pregnancy ended in weeks? (For example 12 weeks gestation, 39 weeks gestation, etc.) __________________ weeks gestation

(N-07) How many fetuses or babies were in this pregnancy? (*Don't read options aloud; please mark* ☒ *one*)
☐ One ☐ Twins ☐ Triplets ☐ More than Triplets
☐ Unknown

(N-06) How did this pregnancy end?
Don't read options aloud; please mark ☒ *one*
☐ Live birth ☐ Miscarriage ☐ Ectopic pregnancy
☐ Stillbirth ☐ Molar pregnancy ☐ Abortion
☐ Other, describe ________________________________
→*If the outcome was* ***not a live birth****, skip to the beginning of N, enter her next "event while avoiding pregnancy." If this is her last "event while avoiding pregnancy," then skip to Section P.*

(N-07) What was (were) the baby's(ies') name(s), sex and birth weight?

	First (given name)	Middle	Last (surname	Birth weight (pounds/ounces or grams)	Sex	Hospital stay of 7 days or more
Baby 1					☐ Male ☐ Female	☐ Yes<br>☐ No<br>☐ Unsure
Baby 2					☐ Male ☐ Female	☐ Yes<br>☐ No<br>☐ Unsure
Baby 3					☐ Male ☐ Female	☐ Yes<br>☐ No<br>☐ Unsure

(N-09) Did you ever breastfed this/these baby(ies)?
☐ Yes ☐ No

(N-10) How long did you breastfed this/these baby(ies)? (in months)
________________ months
*If there were multiple babies with different answers, record the answer for the baby with the **longest** time breastfeeding.*

(N-11) How old was/were this baby(ies) when (s)he first ate something other than breastmilk or water?
________________ months
*If there were multiple babies with different answers, record the answer for the **last** baby to have something besides breastmilk or water.*

If this is her last event that happened while avoiding pregnancy, skip to section P.

SECTION P

Finally, I have a few questions about participating in studies of treatment for infertility. Your answers to these questions may help us design future studies. To answer these questions, please look back to the time that you first were considering treatment for infertility.

If she objects that she never considered treatment, then ask her to look back to the time that she first realized she was having difficulty getting pregnant, or at least 6 months from when she didn't get pregnant despite regular intercourse without contraception.

(P-1) At that time, would you have been willing to participate in a research study that involved receiving fertility treatment, at no cost to you?

☐ Yes ☐ Maybe ☐ No

If YES or MAYBE, proceed to Q P-3.

(P-2) (*If NO to Q P-1)* Can you tell my why you would not have wanted to participate in a study of fertility treatments?

____
____
____

(P-3) Under which of the following conditions would you have been willing to participate in a study involving fertility treatment? (Note all that apply)

a. If one of the treatments you might receive would have been advice regarding lifestyle?
   i. If no, please explain why
b. If one of the treatments you might have received would have been education about the most fertile days of your menstrual cycle, in order to learn what days you are most likely to get pregnant?
c. If one of the treatments you might have received had included alternative treatments such as herbs or acupuncture?
d. If one of the treatments you might have received had included medical intervention, such as medication, to increase the chances of pregnancy from natural intercourse?
e. If one of the treatments you might have received would have been artificial insemination?
f. If one of the treatments you might have received would have been in vitro fertilization (IVF)?

2. In a randomized study, participants do not choose their individual treatments and the type of treatment received is entirely left up to chance. Would you have been willing to participate in a randomized research study on two different fertility treatments, where which treatment you got was determined entirely by chance?
   a. If NO: Can you tell me why you would not have wanted to participate in a **randomized** study of two different fertility treatments (or why you would be unsure about participating in a **randomized** study of two different fertility treatments?)
   ____
   ____
   ____
   END HERE
   b. IF yes or maybe, proceed to question 3

3. Which of the following would have increased the likelihood of your participation in a **randomized** study of two different fertility treatments?
   a. Getting free treatment

b. The possibility of receiving more involved treatment after participating in the study for some time [if necessary, clarify that "more involved" means more medications, procedures and interventions]
    i. As part of a study, **how long** would you have been willing to receive less involved treatment before moving on to more involved treatment?
c. Getting paid for participation in addition to the treatment received
d. Is there anything else that would have increased your likelihood of participation in a randomized study of two different fertility treatments?
____
____
____

4. How long would you have been willing to participate in a **randomized** study of fertility treatment?
    a. Clarify response in terms of months and years

5. Finally, would you participate in a randomized study of two different fertility treatments if you had to pay for the treatments yourself?

6. Do you have any other comments about participating in randomized studies of fertility treatments?
____
____
____

SECTION Q: Other Sources of Information

(Q-01) For the written questionnaire or this interview, did you review any copies of medical records from doctors?
☐ Yes ☐ No

(Q-02) For the written questionnaire or this interview, did you review any of your own summaries, diaries, calendars, or journals about your fertility experiences?
☐ Yes ☐ No

SECTION R: Future Studies
R-01: May we contact you in the future for additional information regarding your fertility experiences?
☐ Yes ☐ No
